# The virulence potential of *Prototheca* microalgae explored by *in vitro* infection model

**DOI:** 10.3389/fcimb.2026.1832882

**Published:** 2026-06-02

**Authors:** Angelika Proskurnicka, Patryk Mazur, Bohdan Paterczyk, Agnieszka Kwiatek, Julita Nowakowska, Mateusz Iskra, Jacek Bielecki, Robin May, Tomasz Jagielski

**Affiliations:** 1 Department of Medical Microbiology, Institute of Microbiology, Faculty of Biology, University of Warsaw, Warsaw, Poland; 2 Imaging Laboratory of Electron and Confocal Microscopy, Faculty of Biology, University of Warsaw, Warsaw, Poland; 3 Department of Molecular Virology, Institute of Microbiology, Faculty of Biology, University of Warsaw, Warsaw, Poland; 4 Institute of Microbiology and Infection and School of Biosciences, College of Life and Environmental Sciences, University of Birmingham, Edgbaston, Birmingham, United Kingdom

**Keywords:** adhesion, adherence, *in vitro* model, internalization, *Prototheca* spp., protothecosis, keratinocytes, macrophages

## Abstract

**Introduction:**

The genus *Prototheca* comprises achlorophyllous, obligately heterotrophic algae. To date, six species (*P. blaschkeae*, *P. bovis*, *P. ciferrii*, *P. cutis*, *P. miyajii*, and *P. wickerhamii*) have been recognized as pathogens of vertebrates, including humans, cattle, and companion animals. Protothecal infections are an emerging global concern, as reported cases continue to rise, especially in the veterinary sector. However, knowledge of the pathogenesis of the disease, including hostpathogen interactions remains rudimentary. Therefore, this study was conceived to investigate these interactions using an *in vitro* model, focusing on three clinically relevant species: *P. bovis*, *P. ciferrii*, *P. wickerhamii*, and, for the first time, the environmental species *P. stagnora*.

**Methods:**

Adhesion and internalization were assessed in murine keratinocytes (Kera 308) and macrophages (Raw 264.7) using co-culture assays and confocal microscopy. The findings were further corroborated by scanning and transmission electron microscopy.

**Results:**

Bovine-associated species (*P. bovis* and *P. ciferrii*) efficiently adhered to macrophages (adhesion rate [AR], 29–83.8%) and were internalized at high levels (internalization rate [IR], 87.5–97.3%). The human-associated *P. wickerhamii* also interacted with macrophages, albeit to a somewhat lesser extent (AR, 22.6–35.8%; IR, 86.8–94.9%). Similar trends were observed in keratinocytes, although overall adhesion and internalization were less efficient (7.6–19.4% and 0–62%, respectively). In contrast, *P. stagnora* exhibited poor adhesion to both cell types (0.3–5.1%) with internalization occurring only in macrophages. Electron microscopy-assisted analyses revealed that pathogenic *Prototheca* species persist intracellularly, residing structurally intact within the macrophage cytoplasm.

**Discussion:**

In summary, the results of this study provide evidence for the invasive potential of pathogenic *Prototheca* species toward host cells and point to cytosolic escape as a plausible mechanism of intracellular survival of the pathogen. Collectively, this study expands our understanding of *Prototheca* pathogenicity and highlights interspecies variation as a key determinant of virulence.

## 1 Introduction

*Prototheca* species are unicellular, achlorophyllous, yeast-like microalgae broadly distributed across moist and organic-rich environments (Jagielski and Lagneau, 2007). Although generally saprophytic, these microorganisms can act as opportunistic pathogens in both humans and animals and cause various infections collectively referred to as protothecosis. Of the 18 currently recognized *Prototheca* species, six, namely *P. blaschkeae*, *P. bovis*, *P. ciferrii*, *P. cutis*, *P. miyajii*, and *P. wickerhamii*, have been implicated as etiological agents of disease (Jagielski and Lagneau, 2007; Lass-Flörl and Mayr, 2007; Todd et al., 2012; Jagielski et al., 2019a; Masuda et al., 2021; Jagielski et al., 2025). Whereas *P. bovis* accounts for the vast majority of bovine mastitis cases, the most prevalent form of animal protothecosis, *P. wickerhamii* is the primary cause of human infections, which typically present as cutaneous, articular or systemic manifestations (Todd et al., 2012; Jagielski et al., 2019b).

Protothecosis has long been regarded as a condition of minor clinical relevance in veterinary and human medicine (Todd et al., 2018). However, recent years have witnessed a growing number of cases worldwide, which is likely attributable to enhanced clinical and laboratory awareness and improved diagnostic tools, but also to an increasing population of elderly individuals and those suffering from different forms of immunosuppression (Lass-Flörl and Mayr, 2007; Todd et al., 2012; Khan et al., 2018; Todd et al., 2018). In the veterinary sector, of particular concern has been bovine mammary protothecosis, which incurs heavy economic losses to the global dairy industry each year (Todd et al., 2018; Shave et al., 2021; Libisch et al., 2022). In the absence of safe and effective drugs or vaccines, culling remains the only option for controlling the epidemic. Likewise, there are no standardized treatment protocols for human protothecosis, leaving clinicians to rely on empirical therapy, which, in disseminated cases, carries a poor prognosis with mortality exceeding 50% (Todd et al., 2012; Todd et al., 2018). The refractoriness of *Prototheca* species to most drug regimens currently available, along with diagnostic misses or delays, significantly compromise the proper clinical management of the *Prototheca* disease (Lass-Flörl and Mayr, 2007; McMullan et al., 2011; Todd et al., 2012; Todd et al., 2018). This emphasizes the challenge that protothecosis poses to healthcare providers.

The long-standing scientific neglect of the issue of protothecosis has reverberated in the scant literature in the field. This is readily apparent in the very poor understanding of the pathogenesis of the disease, including host-pathogen interactions and host-imposed stress conditions. Among the few studies exploring the interface between *Prototheca* and host cells, virtually all have focused on algal adherence and cellular entry (**Table 1**). These studies have demonstrated that *Prototheca* algae vary markedly in their capacity to adhere to, invade, and persist within host cells, and that these variations occur at both species and strain levels (Deng et al., 2016; Shahid et al., 2017b; Yang et al., 2020; Haider et al., 2023; Shave et al., 2025). For instance, *P. bovis* exhibited considerably higher adhesion efficiency than *P. ciferrii* or *P. wickerhamii* across various cell lines, including bovine mammary epithelial cells, murine macrophages, and human monocyte-derived macrophages (Deng et al., 2016; Shahid et al., 2017b; Haider et al., 2023). P*. bovis*, and other cattle-associated species (*P. ciferrii* and *P. blaschkeae*) were more susceptible to phagocytosis by human and murine macrophages than human-associated species (*P. cutis*, *P. miyajii*, *P. wickerhamii*) *(*Shave et al., 2025). Also, clinical isolates of *P. ciferrii* showed enhanced uptake and survival inside macrophages compared to an isolate of environmental origin (Yang et al., 2020). Interestingly, whereas both *P. bovis* and *P. ciferrii* were able to induce apoptosis once internalized into murine mammary epithelial cells (Shahid et al., 2017b; Shahid et al., 2020), neither of the two species triggered apoptosis or persisted in murine osteoblasts, suggesting tissue-specific tropism (Shahid et al., 2017b). Moreover, distinct cytokine induction patterns and nitric oxide responses among strains support the hypothesis that both interspecies and intraspecies variation drive differences in immune modulation strategies (Deng et al., 2016; Yang et al., 2020).

**Table 1:** Summary of experimental data on *Prototheca*–host cell interactions – a literature review.

No.	Year^a^	Cell line^b^	*Prototheca* sp.	MOI^c^	Time^d^	Adhesion^e^	Internalization^f^	Ref.
1.	1981	hPMNs	*P. wickerhamii*	1:10	0–2	ND	+	(Phair et al., 1981)
2.	2006	bPMNs	*P. zopfii* ^g^	10:1	0.75	ND	+	(Cunha et al., 2006)
3.	2016	bMECs	*P. bovis* *P. ciferrii*	5x10^4^	0.5; 1; 1.5; 2; 2.5	++ +	ND	(Deng et al., 2016)
4.	2017	bMECs	*P. bovis* *P. ciferrii*	5:1	1; 4; 8; 12; 24	++ +	+ +	(Shahid et al., 2017b)
5.	2017	bMECs	*P. bovis* *P. ciferrii*	5:1	4; 12; 24	+ +	ND	(Shahid et al., 2017a)
6.	2020	bMECs; mMφ (J774)	*P. bovis* *P. ciferrii*	1×10^5^ 5×10^5^	1; 2; 4; 8; 12	ND	++ +	(Shahid et al., 2020)
7.	2020	mMφ (J774A.1)	*P. ciferrii*	5:1	3; 6; 9	ND	+	(Yang et al., 2020)
8.	2023	mMφ (J774A.1; iBMDMs); HMDMs	*P. bovis* *P. wickerhamii*	3:1; 1:1	0–6	ND	++ +/-	(Haider et al., 2023)
9.	2025	mMφ (J774A.1); HMDMs	*P. bovis* *P. blaschkeae* *P. ciferrii* *P. cookei* *P. cutis* *P. miyajii* *P. moriformis* *P. paracutis* *P. tumulicola* *P. wickerhamii* *P. xanthoriae*	3:1; 1:1	1	ND	++ ++ ++ ++ +/– + +/– + +/– +/– ++	(Shave et al., 2025)

^a^ Year of report publication;.

^b^ h(b)PMNs, human (bovine) polymorphonuclear neutrophils; bMECs, bovine mammary epithelial cells; mMφ, murine macrophages; iBMDMs, immortalized bone marrow-derived macrophages; HMDMs, human monocyte-derived macrophages;.

^c^ MOI – multiplicity of infection (ratio of pathogen to infection target); if a numerical value is provided, it refers to the *Prototheca* sp. concentration and is expressed in CFU/mL;.

^d^ Time – time of exposure (hours);.

^e^ A double plus symbol (++) indicates that adhesion was more efficient;.

^f^ Symbols indicate incremental internalization efficiency (++ > + > +/–); based on the calculated phagocytic index, > 40% (++); 11–40% (+), and 0–10% (+/–); in study no. 6, mMφ (J774) were infected only with *P. bovis*; in study no. 8, internalization of the genus *P. wickerhamii* was confirmed only in HMDM cells;.

^g^ According to current *Prototheca* taxonomy, bovine isolates previously classified as *P. zopfii* most probably represent either *P. bovis* or *P. ciferrii*.

This study was conceived to further develop an *in vitro* model of protothecosis to investigate interactions between *Prototheca* algae and murine host cells. The study involved four *Prototheca* species, including three major pathogens of cattle (*P. bovis*, *P. ciferrii*) and humans (*P. wickerhamii*), and, for the first time, *P. stagnora*, a typically saprophytic species. Two murine cell types were used in the model. Keratinocytes were included to reflect the skin environment, as *Prototheca* spp. are commonly associated with cutaneous infections in both humans and animals. Notably, their role in *Prototheca*–host interactions has not previously been investigated. In parallel, macrophages were used as a proxy for the innate immune response. A microplate adherence assay was performed to evaluate the adhesion capacity of *Prototheca* species. Algal internalization was assessed using confocal microscopy and further verified by scanning and transmission electron microscopy.

## 2 Materials and methods

### 2.1 *Prototheca* spp. strains and culture

Four type *Prototheca* sp. strains obtained from the American Type Culture Collection (ATCC) and the Culture Collection of Algae at the University of Göttingen (SAG) were used in the study. Included in this number were three pathogenic (*P. bovis* SAG 2021, *P. ciferrii* SAG 2063, *P. wickerhamii* ATCC 16529) and one saprophytic (*P. stagnora* ATCC 16528) species. The strains were cryopreserved using Viabank Storage Beads (MWE Medical Wire, United Kingdom) at -80 °C. Prior to experiments, strains were revived and subcultured on Sabouraud Dextrose Agar (SDA; Biomaxima, Poland) and incubated at 37 °C for 72 h under aerobic conditions.

### 2.2 Murine cell culture

Cell lines of murine macrophages (Raw 264.7; ATCC, USA) and keratinocytes (Kera 308; CLS, Germany) were cultured in Dulbecco’s Modified Eagle’s Medium (DMEM; Sigma-Aldrich, USA) supplemented with 10% fetal bovine serum (FBS; Sigma-Aldrich, USA), 2-mM L-glutamine (Gibco, USA), and 100-U/mL penicillin, streptomycin, amphotericin B solution (Capricorn Scientific, Germany), and incubated at 37 °C in a humidified atmosphere containing 5% CO_2_.

### 2.3 *In vitro* infection

**Figure 1:**
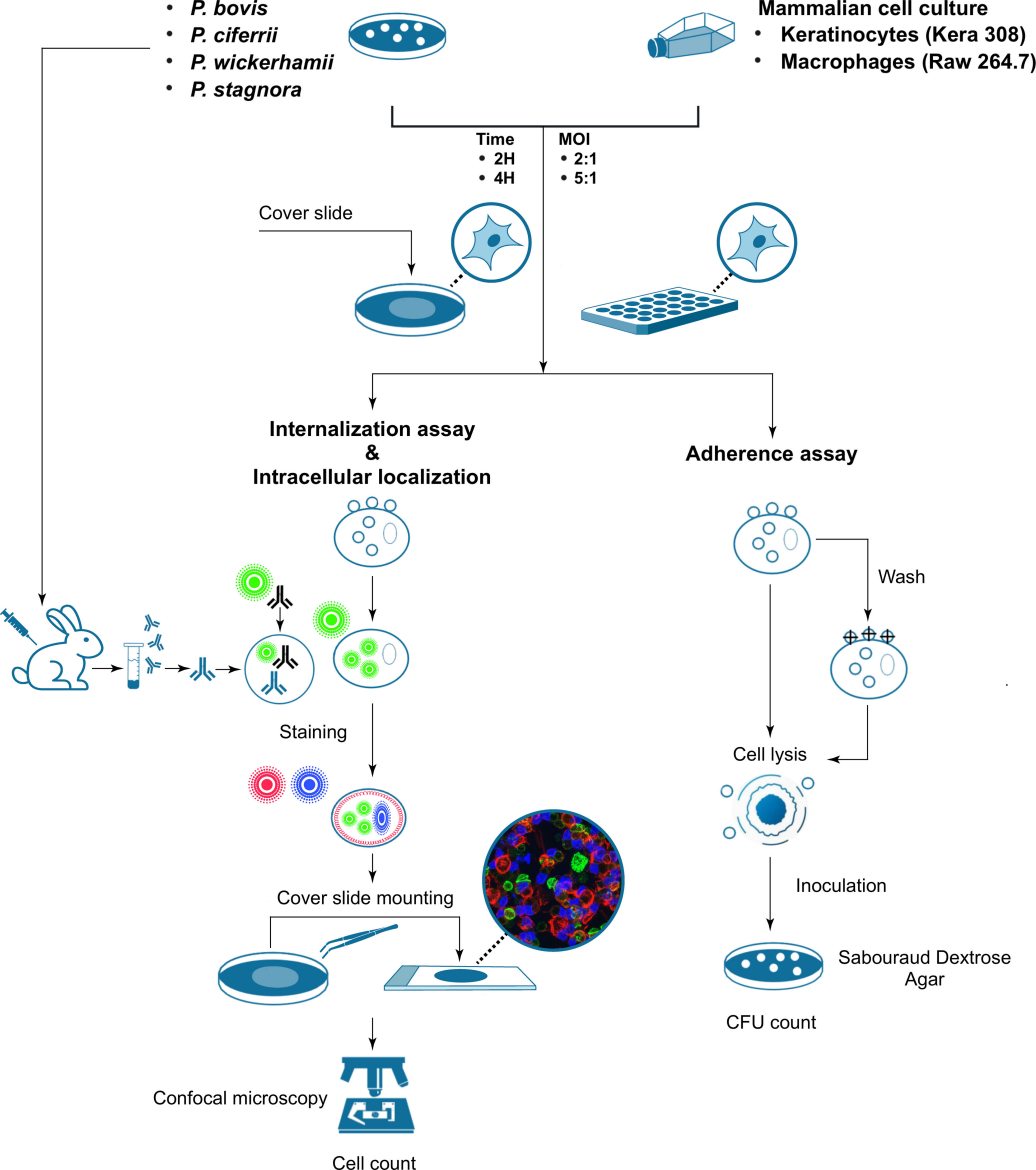
Schematic overview of the experimental design and workflow of the study.

For experimental infection, murine cells were seeded at 1x10^5^ cells/well in 24-well plates (adherence assay) or at 5x10^5^ cells/dish in Ø35-mm Petri dishes containing sterile glass coverslips (microscopic evaluation) with antibiotic-free medium and incubated as described above for 72 h until confluent monolayer was formed. For each experiment, two control wells/dishes were run in parallel to determine the mean number of host cells. Cells from the controls were first detached using TrypLE Express Enzyme (Gibco, USA), centrifuged at 2000 rpm for 5 min at room temperature (RT), and counted using an automated cell counter (ADAM-MC2, NanoEnTek, South Korea). The mean host cell numbers were used to calculate the number of *Prototheca* sp. cells required to achieve the desired multiplicity of infection (MOI) of 2:1 or 5:1 (i.e., two/five algal cells per one mammalian cell). The algal suspensions were prepared as follows. A loopful of colonies was suspended in 10 mL of medium dedicated for murine cell culture. The number of *Prototheca* sp. cells in the solution was determined using an improved Neubauer counting chamber. The suspension was then diluted to achieve the required concentration corresponding to a MOI of 2:1 or 5:1 and used to expose the host cells. After removing the culture medium, the murine cells were washed three times with phosphate-buffered saline (PBS; pH=7.4). The algal suspension was then added to the host cell culture, at a volume of 1 mL per well or 2 mL per Petri dish, and incubated at 37 °C in a humidified 5% CO_2_ atmosphere for 2 or 4 h prior to further analysis. A schematic diagram of the experimental *in vitro* infection is presented in **Figure 1**.

### 2.4 Adherence assay

After incubation of the co-cultures in 24-well plates, the supernatants from each well were removed, and the wells were washed three times with PBS to remove non-adherent algal cells. Subsequently, murine cells were lysed with 0.1% Triton-X solution (Sigma-Aldrich, USA) to release adherent *Prototheca* cells. Thus, the calculated adhesion rate represents the total number of host cell–associated *Prototheca* cells (both surface-adhered and internalized) released upon host cell lysis. To determine the total number of algae (both adherent and non-adherent), the supernatant from each well was retained and combined with the lysate. No PBS washing was performed after lysis to avoid loss of non-adherent cells.

A tenfold dilution of each suspension was plated onto SDA plates and incubated aerobically at 37 °C for 72 h to enumerate colony-forming units (CFU) per well. Adherence rate (AR) of *Prototheca* spp. was defined as:


$$\text{AR }[\%]=\;\frac{\text{no}.\text{ of adhered algae cells }(\text{lysate})}{\text{total no}.\text{ of algae cells }(\text{supernatant and lysate})}\text{x }100$$

For each experimental condition, the mean AR was based on 10 replicates from 3 independent experiments.

### 2.5 Internalization assay

The internalization assay was performed after 2 or 4 h of incubation with *Prototheca* sp. cells at MOI of 2:1 or 5:1. Upon completion of the infection period, murine cells were washed three times with PBS and fixed in 3.7% formaldehyde (Sigma-Aldrich, USA) for 10 min at 37 °C. Unless otherwise stated, all subsequent staining procedures were carried out at RT and preceded by three PBS washes. Cells were permeabilized with 0.1% Triton-X for 10 min, and then treated with blocking solution containing 0.1% bovine serum albumin (BSA) and 3% non-fat dry milk for 2 h. *Prototheca* cells were visualized by overnight incubation with primary rabbit anti-*Prototheca* IgG (1.06–1.68 μg/mL; Davids Biotechnologie, Germany), which were generated against the individual *Prototheca* strains used in this study, ensuring strain-specific recognition of the analyzed isolates. Detection was subsequently performed using Alexa Fluor 488-conjugated goat anti-rabbit IgG (1 µg/mL; Abcam, UK). For immunostaining of cellular actin and nuclei, cells were incubated with TRITC-conjugated phalloidin (0.77 µM/mL; Tocris Bioscience, UK) for 40 min at 37 °C, and Hoechst 33258 (0.5 µg/mL; Sigma-Aldrich, USA) for 3 min at 37 °C, respectively. Coverslips were subsequently mounted onto glass microscope slides using Dako Fluorescence Mounting Medium (Agilent Technologies, Denmark). Imaging was performed using A1R MP Confocal System coupled to a TiE Inverted Microscope (Nikon, Japan) equipped with Plan APO VC 60x/1.4 oil objective. The excitation and emission settings for each fluorescence channel were as follows: nuclei (blue channel), Ex 404 nm/Em 425–475 nm; *Prototheca* sp. cells (green channel), Ex 488 nm/Em 500–550 nm; cytoskeleton (red channel), Ex 561 nm/Em 570–620 nm.

*Prototheca* spp. and host cells were quantified using 3D scanning of infected cell cultures. Image stacks were acquired at 1 µm intervals along the Z-axis, allowing visualization of the spatial relationships between *Prototheca* sp. cells, cytoskeletal actin, and nuclei, which served as markers of the host cells’ internal architecture. Host cell counts were determined based on nuclear staining.

Internalization rate (IR) of *Prototheca* spp. was defined as:


$$\text{IR }[\%]=\frac{\text{no}.\text{ of algal cells inside the host cells}}{\text{total no}.\text{ of visualised algal cells }}\text{x }100$$

The internalization assay was conducted in two independent replicates (i.e. two microscope slides) for each experimental condition. For each slide, at least 10 randomly selected microscopic fields were analyzed. Detailed image analysis was performed using Nikon NIS-Elements 4.10 software.

### 2.6 Transmission electron microscopy imaging

The results of the internalization assay were further verified using electron microscopy, with both transmission (TEM) and scanning (SEM) techniques employed to complement confocal imaging and provide a detailed assessment of *Prototheca*-host cell interactions.

As the first step in TEM analysis the infected cells were fixed in 2.5% glutaraldehyde solution (Serva, Germany) in 0.1 M cacodylate buffer (Serva, Germany) for 24 h, RT. After fixation, the samples were washed three times with cacodylate buffer, and subsequently post-fixed in 1% osmium tetroxide (Sigma-Aldrich, USA) for 2 h, followed by three washes with double-distilled water. The samples were then dehydrated through a graded alcohol series and then acetone, 15 min per step. Samples were embedded in Epon resin (Serva, Germany) and 70-nm slices were cut with a diamond knife (Diatome, Switzerland) on an RMC MTX ultramicrotome (Boeckeler Instruments, USA). Sections were mounted on copper grid and stained with 2% uranyl acetate (Serva, Germany) and lead citrate (Sigma-Aldrich, USA). Imaging was performed using a LIBRA 120 transmission electron microscope (Carl Zeiss, Germany), at 120keV, and images were captured with a Slow-Scan CCD camera (ProScan, Germany).

### 2.7 Scanning electron microscopy imaging

SEM analysis was carried out on glass coverslips containing murine cells co-cultured with *Prototheca* sp. cells. Once the infection period was complete, cells were fixed in 2.5 % glutaraldehyde in 0.1 M cacodylate buffer (Serva, Germany) for 16 h, RT. The samples were then washed three times with cacodylate buffer over 30 min and post-fixed in 1% osmium tetroxide (Sigma-Aldrich, USA) for 2 h, RT. Subsequently, cells were rinsed three times with distilled water over 30 min before dehydration in an ascending ethanol series (Poch, Poland). Samples were dried by the Critical Point Drying System (Polaron, UK), mounted on aluminum stubs, and sputter-coated with gold (Polaron SC7620, UK). SEM images were acquired and analyzed with LEO-32 software (Carl Zeiss, Germany). All electron microscopy (TEM and SEM) analyses were performed exclusively after 4 h of incubation with algal cells at a MOI of 2:1.

### 2.8 Statistical analysis

Statistical analyses were performed in GraphPad Prism 10.4.2 software (GraphPad, USA). Before performing tests of group comparisons, assumptions of normality, homogeneity of variances and lack of autocorrelation were checked. Levels of significance were determined by U-Mann-Whitney, Kruskal-Wallis with Dunn’s *post hoc* test. For the internalization assay, Fisher’s exact test was used. Differences were considered statistically significant when *p* < 0.05 (* *p* < 0.05; ** *p* < 0.01; *** *p* < 0.001; **** *p* < 0.0001).

## 3 Results

### 3.1 Adherence assay

**Figure 2:**
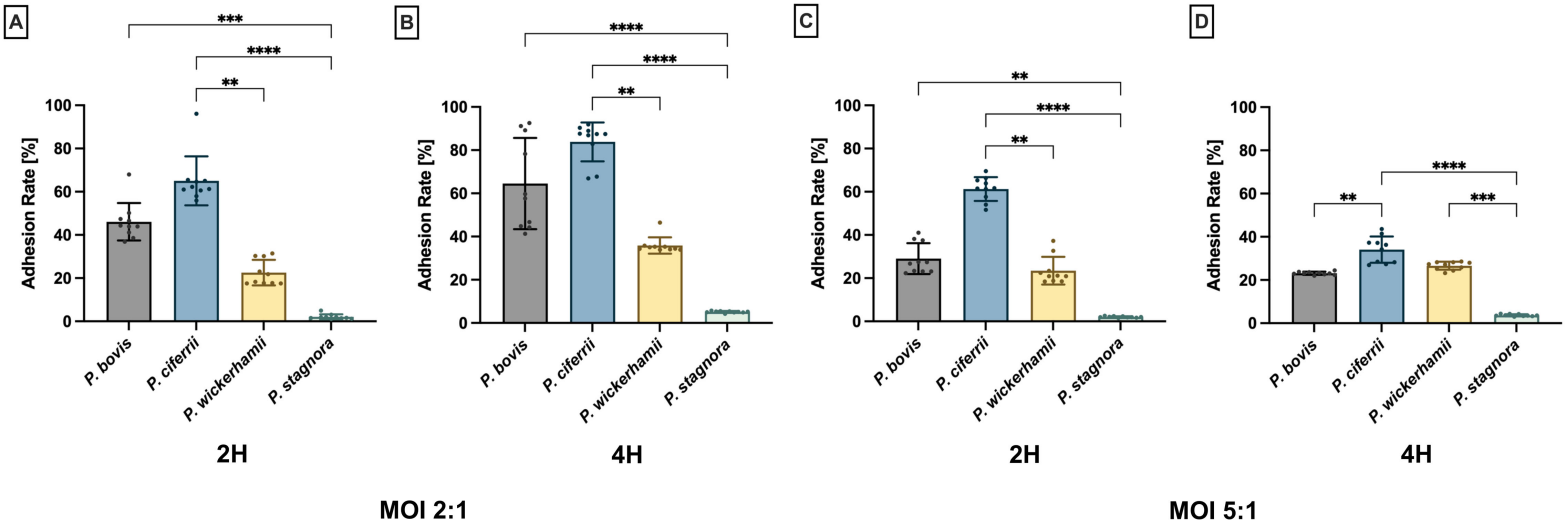
Adhesion rate (%) of Prototheca species to RAW 264.7 macrophages at different multiplicities of infection (MOI 2:1 [**(A, B)** and 5:1 **(C, D)**] and incubation times [2 h **(A, C)** and 4 h **(B, D)**]. Statistical significance was assessed using the Mann–Whitney U test and the Kruskal-Wallis test with Dunn’s post hoc test (** *p* < 0.01; *** *p* < 0.001; **** *p* < 0.0001).

**Figure 3:**
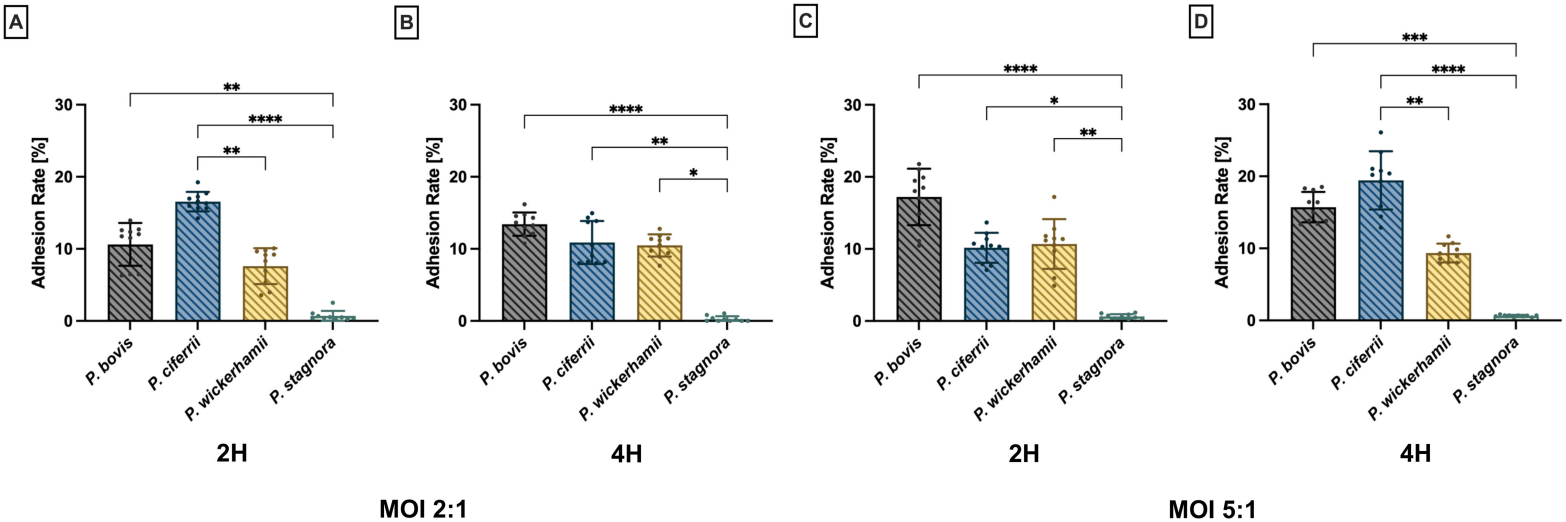
Adhesion rate (%) of Prototheca species to Kera 308 keratinocytes at different multiplicities of infection [MOI 2:1 **(A, B)** and 5:1 **(C, D)**] and incubation times [2 h **(A, C)** and 4 h **(B, D)**]. Statistical significance was assessed using the Mann–Whitney U test and the Kruskal-Wallis test with Dunn’s post hoc test (* *p* < 0.05; ** *p* < 0.01; *** *p* < 0.001; **** *p* < 0.0001).

The four *Prototheca* species exhibited distinct adherence patterns, with adherence rate (AR) varying significantly across both cell lines (**Figures 2**, **3**). In macrophages, the highest AR was recorded for *P. ciferrii*, reaching 83.8% at an MOI of 2:1, after 4 h of exposure. Less efficient adherence was observed for *P. bovis* and *P. wickerhamii*, with the highest ARs of 64.6% and 35.8%, respectively (*p* < 0.05). *P. stagnora* showed marginal adherence to macrophages, with AR values not exceeding 6% under any conditions (**Figures 2A–D**).

As for keratinocytes, the ARs for all species were on average about 40% lower than those observed for macrophages (*p* < 0.0001) (**Figures 2**, **3**). *P. ciferrii* and *P. bovis* showed the highest and comparable ARs, with values ranging from 10.2–19.4% and 10.6–17.2%, respectively, depending on MOI and incubation time (*p* < 0.05). Contrastingly*, P. wickerhamii* produced lower ARs, relatively invariant across experimental conditions, ranging 7.6–10.7% (*p* < 0.05). *P. stagnora* exhibited only minimal adhesion, with ARs remaining below 1% under all conditions (*p* < 0.05) (**Figures 3A–D**).

Overall, all species exhibited markedly higher adhesion to macrophages than keratinocytes. Both MOI and incubation time affected AR, yet neither produced a consistent increase in adherence, and most observed differences were not statistically significant. The strongest effect was seen with macrophages, where adhesion was on average about 60% higher at a lower MOI (2:1) (*p* < 0.001) (**Figure 2**). By comparison, in keratinocytes, MOI and incubation time had only minor effects on adhesion (*p*>0.05) (**Figure 3**).

### 3.2 Internalization assay

**Figure 4:**
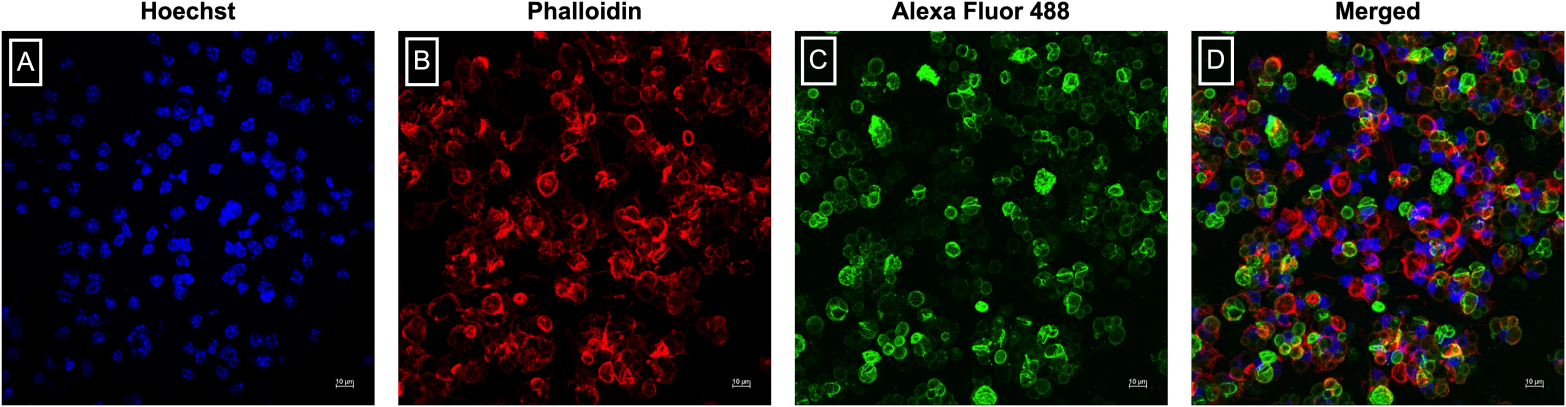
Confocal microscopy of raw 264.7 macrophages infected with *P. ciferrii* (MOI = 5:1, 4 h), showing individual fluorescence channels **(A–C)** and the merged image **(D)**. The spatial relationship between cellular components enabled assessment of the intracellular vs. extracellular localization of algal cells. Nuclei (blue), actin cytoskeleton (red), and *Prototheca* cells (green). Scale bar: 10 µm.

**Figure 5:**
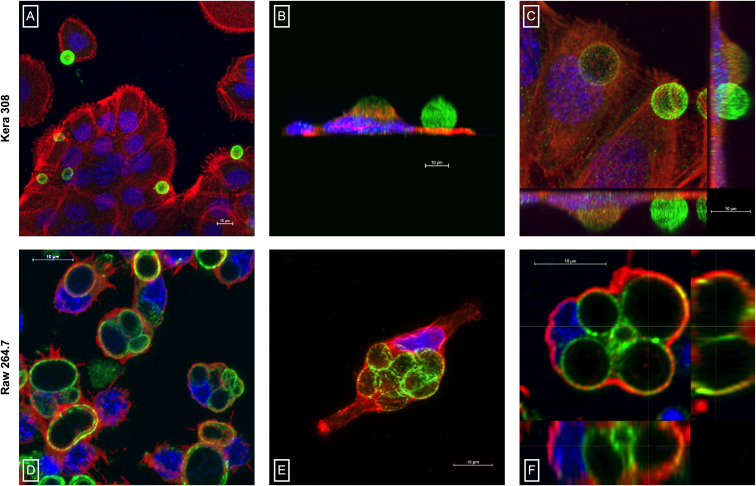
Details of the internalization process as exemplified by *P. bovis* entering Kera 308 keratinocytes **(A–C)** and Raw 264.7 macrophages **(D–F)**. Algal cells at different stages of internalization **(A)**; Two algal cells, one attached to the cell surface and the other enclosed by the actin cytoskeleton indicating intracellular penetration, shown in lateral **(B)** and orthogonal **(C)** views; Algal cells surrounded by the actin cytoskeleton and associated host cell morphological distortion, depicted in a cross-sectional projection **(D)**; A macrophage containing multiple algal cells in a planar **(E)** and cross-sectional **(F)** projections. Nuclei (blue), actin cytoskeleton (red), and *Prototheca* cells (green). Scale bar: 10 µm.

Interpretation of the internalization assay required a clear determination of the intracellular versus extracellular localization of algal cells, based on meticulous confocal microscopy analysis of the spatial colocalization between the pathogen and host cell structures (**Figures 4**, **5**).

**Figure 6:**
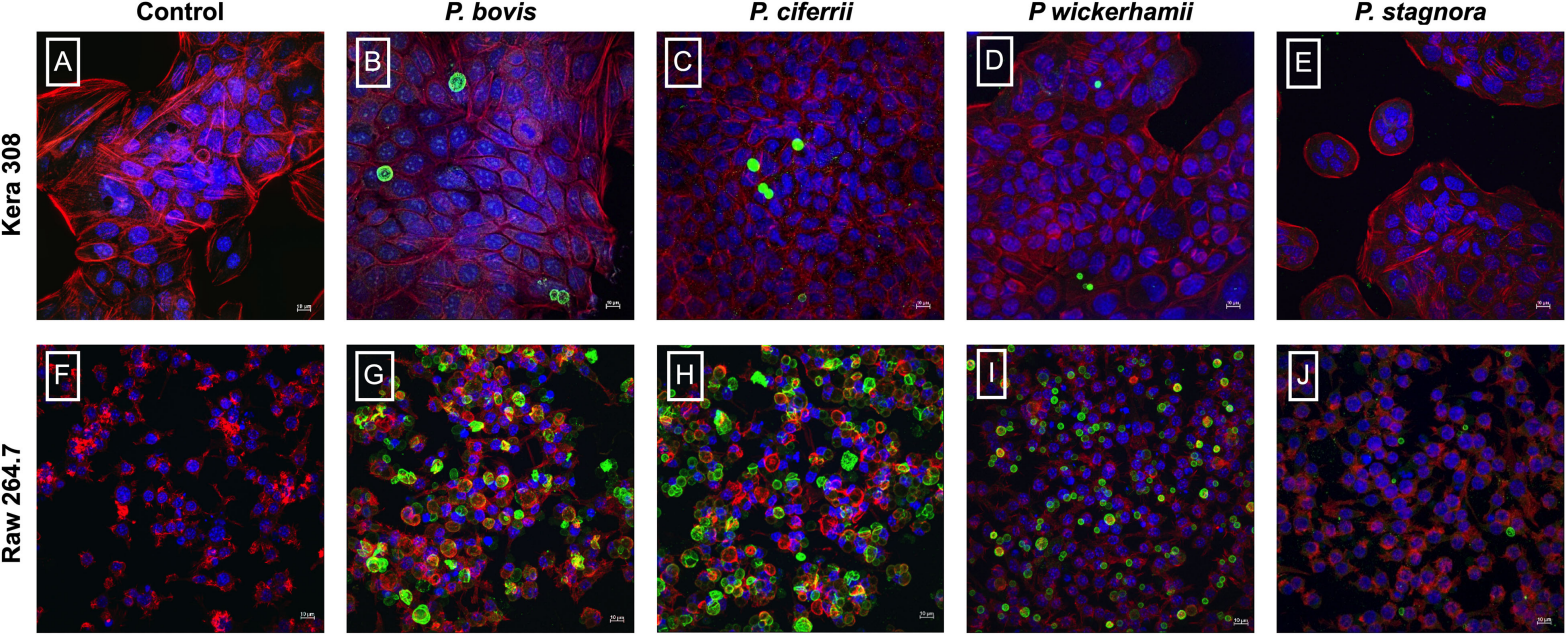
Confocal microscopy images illustrating the internalization of *Prototheca* algae to Kera 308 keratinocytes **(A–E)** and Raw 264.7 macrophages **(F–J)** (MOI 5:1, 4 h). Species-dependent differences in the extent of algal internalization can be observed. Nuclei (blue), actin cytoskeleton (red), and *Prototheca* cells (green). Scale bar: 10 µm.

**Figure 7:**
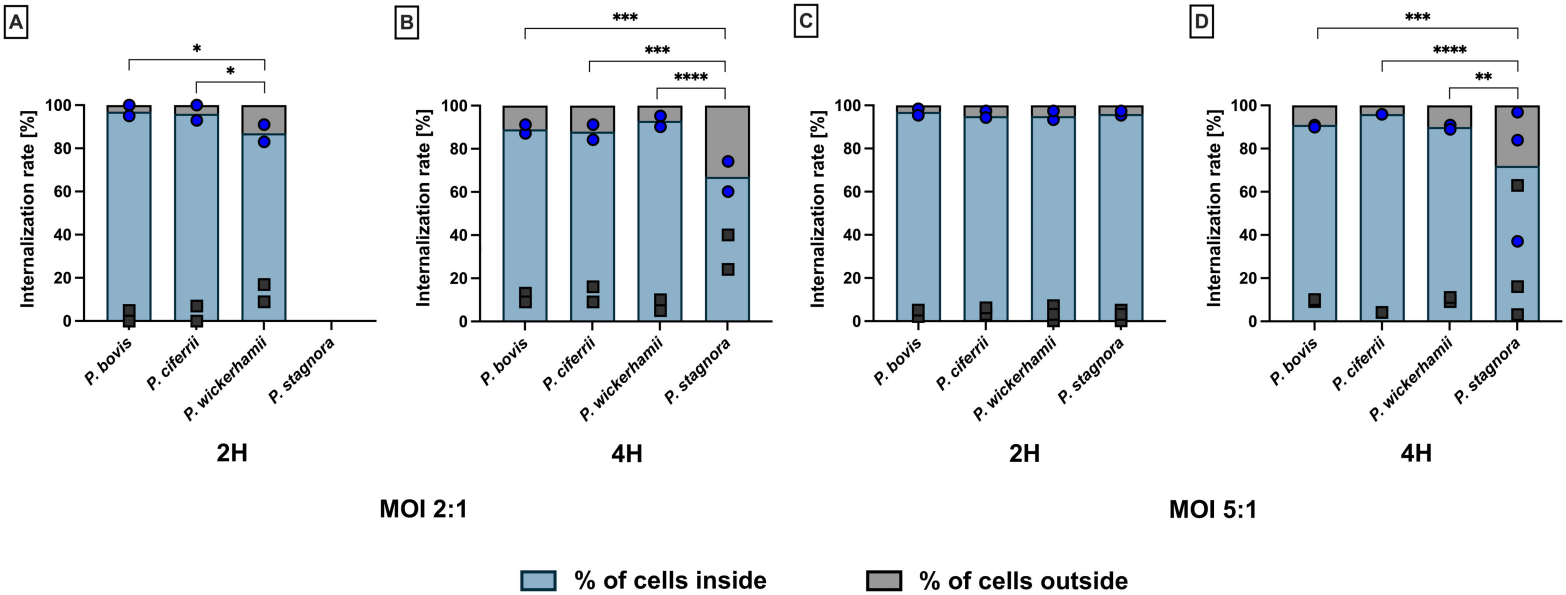
Internalization rate (%) of *Prototheca* algae to Raw 264.7 macrophages at different multiplicities of infection [MOI 2:1 **(A, B)** and 5:1 **(C, D)**] and incubation times [2 h **(A, C)** and 4 h **(B, D)**]; internalized cells are indicated by dots (●) and non-internalized cells by square markers (■). Statistical significance was assessed using Fisher’s exact test (* *p* < 0.05; ** *p* < 0.01; *** *p* < 0.001; **** *p* < 0.0001).

**Figure 8:**
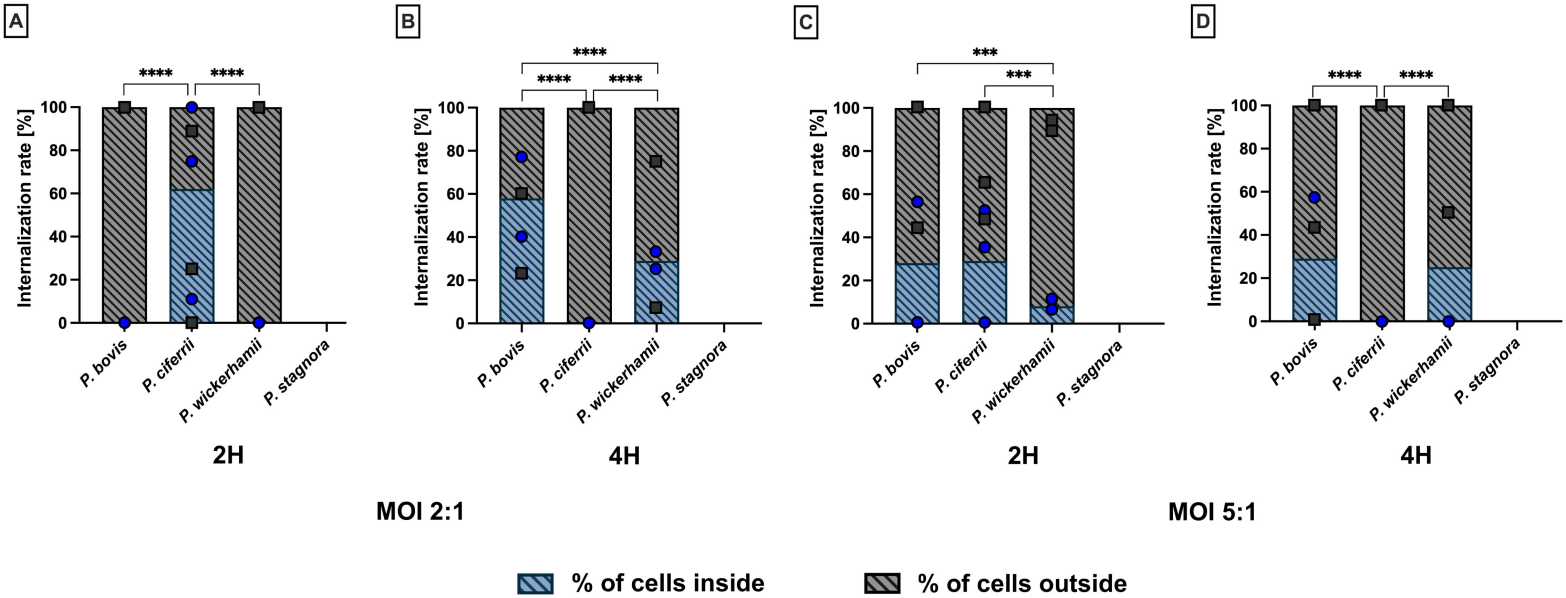
Internalization rate (%) of *Prototheca* algae to Kera 308 keratinocytes at different multiplicities of infection [MOI 2:1 **(A, B)** and 5:1 **(C, D)**] and incubation times [2 h **(A, C)** and 4 h **(B, D)**]; internalized cells are indicated by dots (●) and non-internalized cells by square markers (■). Statistical significance was assessed using Fisher’s exact test (*** *p* < 0.001; **** *p* < 0.0001).

In macrophages, the internalization rate corresponds functionally to the phagocytic index, as algal uptake by these professional phagocytes most likely reflects phagocytosis. Among tested species, *P. bovis*, *P. ciferrii*, and *P. wickerhamii* exhibited similarly high IRs, consistently exceeding 86% across all experimental conditions (*p* > 0.05) (**Figures 6F–J**, **7A–D**). In contrast, the saprophytic *P. stagnora* showed lower internalization overall, although IR values varied widely, ranging from 0% to 96.4% depending on MOI and incubation time (*p* < 0.0001) (**Figures 7A–D**). In keratinocytes, *Prototheca* internalization was markedly less efficient compared to macrophages (*p* < 0.0001) (**Figures 6A–E**, **8A–D**). *P. bovis* and *P. ciferrii* showed highly variable uptake, peaking at 58.5% and 62% respectively, yet internalization was absent in several experimental variants (*p* < 0.05). Conversely, *P. wickerhamii* showed consistently low internalization (8.4–29.2%; *p* < 0.01), while *P. stagnora* was not detected inside keratinocytes under any experimental condition (**Figures 8A–D**).

Clear cell-line-dependent differences in the IR were observed, with macrophages demonstrating significantly greater intracellular uptake (86.8–97.3%) compared to keratinocytes (0–62%; *p* < 0.0001) (**Figures 6**–**8**). Unlike adhesion, the experimental parameters appeared to have a more limited impact on the internalization process. Although IR values varied among species (*p* > 0.05) and conditions (*p* > 0.05), no coherent MOI- or time-dependent pattern was evident (**Figures 7**, **8**).

### 3.3 Transmission and scanning electron microscopy imaging

**Figure 9:**
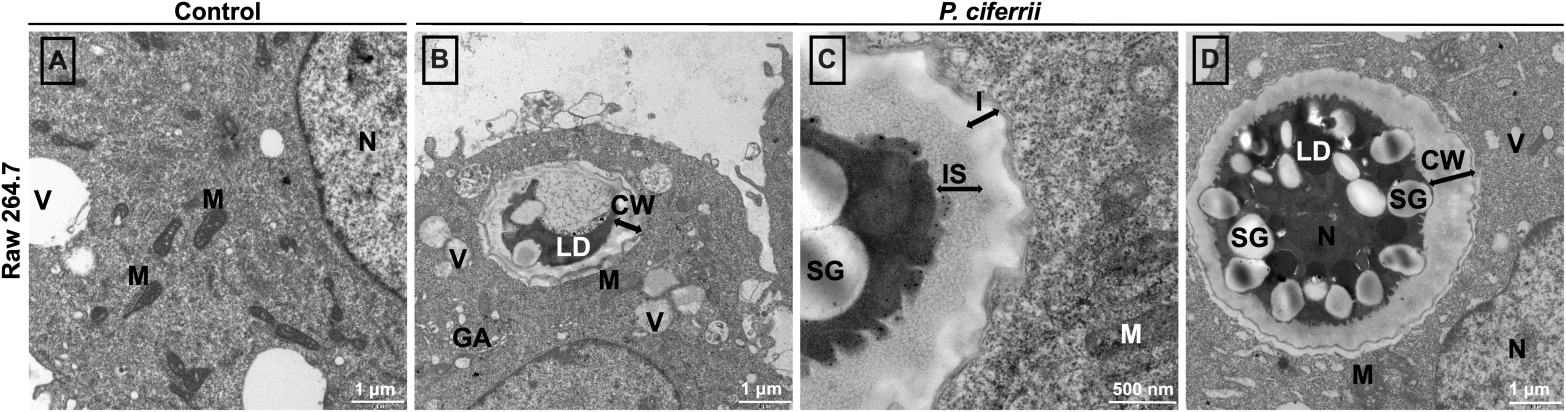
Transmission electron microscopy images illustrating the internalization of *P. ciferrii* to Raw 264.7 macrophages (MOI 2:1, 4 h). An algal cell localized within the macrophage cytoplasm, with an intact cell wall and no signs of structural damage. CW – cell wall; GA – Golgi apparatus; I – intine; IS – interspace; LD lipid droplet; M – mitochondrion; N – nucleus; SG – starch grain; V – vacuole. Scale bars: 1 µm **(A, B, D)**, and 500 nm **(C)**.

TEM analysis revealed no morphological differences between *Prototheca*-infected and uninfected macrophages (**Figure 9A**). Across all four *Prototheca* species, algal cells clearly localized within the cytoplasm, confirming successful uptake by phagocytic cells. Numerous vacuoles were observed in the cytoplasm of *Prototheca*-containing macrophages, whereas the structural integrity of the algal cells remained intact (**Figures 9B–D**). No algal cells were detected within keratinocytes and no intracellular morphological alterations were observed on TEM images.

**Figure 10:**
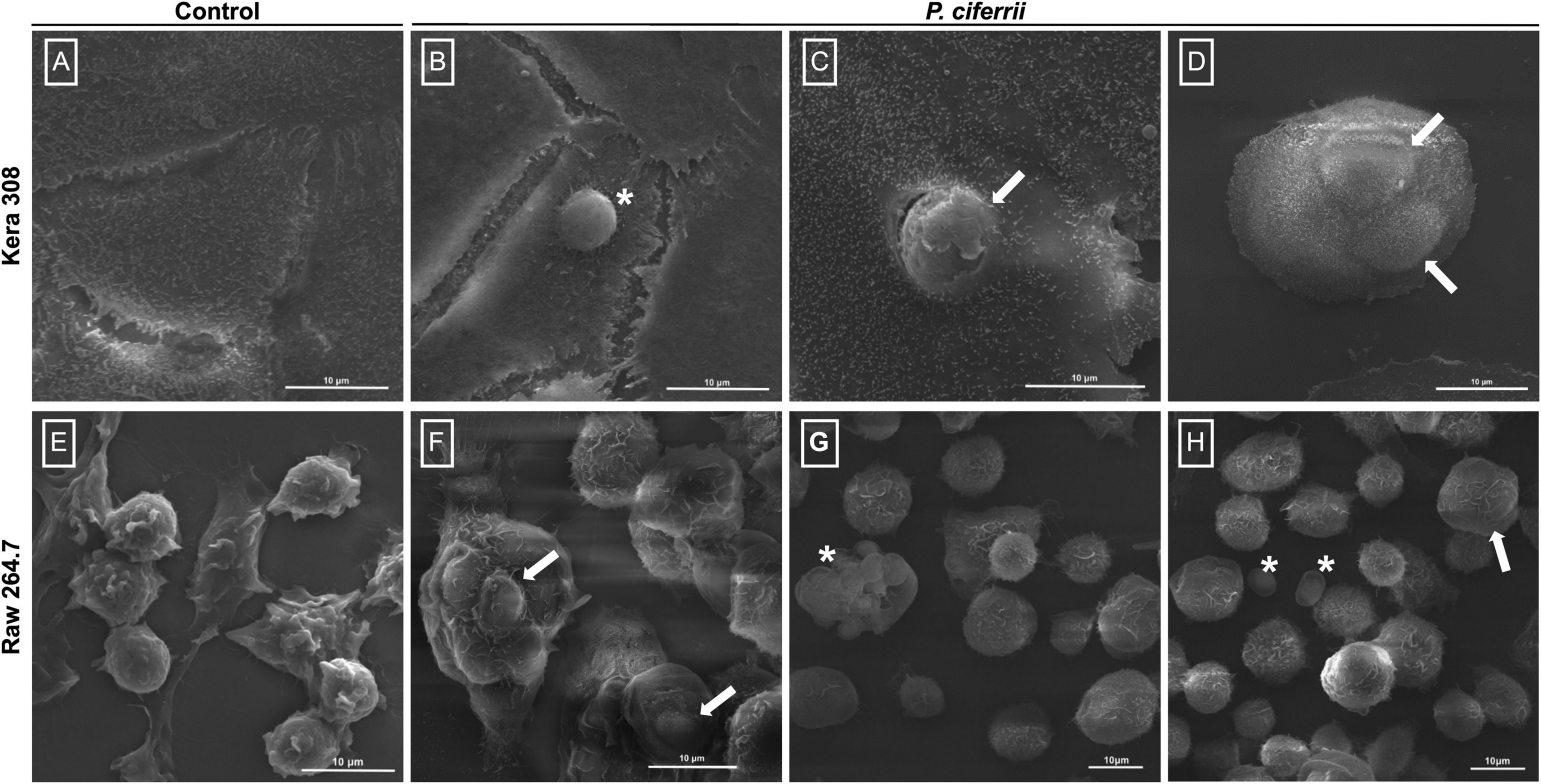
Scanning electron microscopy images illustrating the internalization of *P. ciferrii* to Kera 308 keratinocytes **(A–D)** and Raw 264.7 macrophages **(E–H)** (MOI 2:1, 4 h). Progressive stages of algal entry into keratinocytes, from initial host-cell attachment to complete engulfment **(B–D)**; Macrophages containing multiple algal cells at different developmental stages **(F–H)**; White arrows (➔) indicate internalized algal cells, while extracellular cells are marked with an asterisk (*). Scale bar: 10 µm.

The interactions between *Prototheca* and host cells were further investigated using SEM, which corroborated the confocal microscopy findings. SEM imaging revealed algal cells either adhering to the host cell surface (**Figures 10B, G, H**) or internalized within the cytoplasm of both murine cell lines (**Figures 10C, D, F, H**). Notably, internalization of the algae induced changes in host cell morphology, with both keratinocytes and macrophages adopting a more compact and rounded shape compared to their typical flattened appearance (**Figures A, 10D, F–H**).

## 4 Discussion

The understanding of the mechanisms of pathogenicity in *Prototheca* infections lingers in its infancy. So does the insight into the early host-algae interaction and the subsequent pathogen’s fate in the host milieu. In this study, we systematically examined the adhesion and internalization dynamics of three clinically most relevant species (*P. bovis*, *P. ciferrii*, and *P. wickerhamii*) *(*Lass-Flörl and Mayr, 2007; Todd et al., 2012; Jagielski et al., 2019c; Huilca-Ibarra et al., 2022; Arabe Filho et al., 2023) and one saprophytic species (*P. stagnora*), using murine macrophages and keratinocytes.

Pathogenic species were 20–50 times more efficient in adhering to host cells than the saprophytic one. Likewise, the internalization assay revealed a conspicuous difference between pathogenic and environmental species. While all pathogenic algae were readily internalized, *P. stagnora* showed virtually no internalization in keratinocytes and only very limited uptake by macrophages.

Among the pathogenic species, a clear hierarchical pattern was observed with *P. ciferrii* showing the highest adhesion to macrophages, followed by *P. bovis*, and *P. wickerhamii* (61.1% *vs*. 40.7% *vs*. 27.1%). The three species ranked the same for adhesion to keratinocytes, though the ARs were much lower and the differences between the species less pronounced (14.3% *vs*. 14.2% *vs*. 9.5%). This species-dependent decremental trend also held true for intracellular uptake by macrophages (93.8% *vs*. 93.6% *vs*. 91.2%) and keratinocytes (28.7% *vs*. 22.7% *vs*. 15.7%). Both adhesion and internalization were markedly higher in macrophages than in keratinocytes, reflecting their phagocytic behavior. In contrast, internalization into keratinocytes was sporadic and stochastic, underscoring the low ability of the algae to invade these types of cells. It is important to note that the present study was conducted using a single (type) strain per species and thus does not reflect the extent of intraspecies variability. Both genetic and phenotypic diversity have been reported among *Prototheca* isolates, including differences in virulence, adhesion, and host cell interactions (Zeng et al., 2019; Yang et al., 2020; Guo et al., 2022; Haider et al., 2023). Thus, the differences in adhesion and internalization reported here may, at least in part, be influenced by strain-specific properties.

Previous studies have demonstrated that *Prototheca* spp. can adhere and be internalized by range of mammalian cell types, including mammary epithelial cells (MECs) (Deng et al., 2016; Shahid et al., 2017b; Shahid et al., 2017a; Shahid et al., 2020), macrophages (Shahid et al., 2020; Yang et al., 2020; Haider et al., 2023; Shave et al., 2025), and neutrophils (Phair et al., 1981; Cunha et al., 2006), with efficiency depending on both the algal species and host cell type (Deng et al., 2016; Shahid et al., 2017b; Shahid et al., 2020; Yang et al., 2020; Haider et al., 2023; Shave et al., 2025). In contrast, no adhesion or uptake of *Prototheca* was demonstrated for murine osteoblasts (Shahid et al., 2017b). Both adhesion and internalization into host cells were most efficient with *P. bovis* as the infectious agent (Deng et al., 2016; Shahid et al., 2017b; Shahid et al., 2020; Haider et al., 2023; Shave et al., 2025). Interestingly, cattle-associated species, such as *P. bovis* and *P. ciferrii* were up to 4 times more efficiently phagocytosed by macrophages than other pathogenic species (*P. wickerhamii*, *P. cutis*, *P. miyajii*) *(*Haider et al., 2023; Shave et al., 2025). These differences have been hypothesized to be due to either: (i) inter-species variation in the pathogen-associated molecular patterns (PAMPs) recognition; (ii) the pathogen’s ability to inhibit phagocytosis; (iii) and the uptake pathway exploited (active invasion *vs.* passive phagocytosis) (Shahid et al., 2017a; Shave et al., 2025). The fact that *P. stagnora* was only marginally internalized by macrophages, consistent with previous observations for other saprophytic species (*P. xanthoriae*, *P. moriformis*, *P. tumulicola*) (Shave et al., 2025), and was not internalized at all by keratinocytes, as shown in this study, indicates that the organism can only be passively phagocytosed by phagocytic cells and cannot actively enter into host cells. In this context, the higher internalization rates of pathogenic *Prototheca* species by macrophages, along with the ability of these species to actively penetrate host cells, provide evidence of their genuinely invasive parasitic behavior.

Previous studies showed that pathogenic *Prototheca* strains are more efficiently internalized and persist longer within macrophages, whereas environmental strains induced nitrative stress, via iNOS upregulation, and increased NO production, thereby promoting killing of the pathogen (Yang et al., 2020). In favor of the presence of host immune evasion mechanisms in the pathogenic strains is the observation that killing of *P. wickerhamii* by human neutrophils (PMNs) was affected by serum opsonins (Shahid et al., 2017a). In another study, however, opsonization had a more neutral rather than adverse effect on the phagocytic capacity of human and murine macrophages (Haider et al., 2023). Also, the more delayed and less pronounced reductions in the levels of antioxidant enzymes in MECs infected with *P. ciferrii* compared with *P. bovis*, originally interpreted as a proxy for non-pathogenic phenotype, may in fact point to a strategy allowing the pathogen to circumvent host immunity, specifically, to counteract the detrimental effects of oxidative stress incurred upon infection (Shahid et al., 2017a).

In epithelial models, *P. bovis* adhered with up to 1.5-fold higher efficiency than *P. ciferrii (*Deng et al., 2016; Shahid et al., 2017b). Furthermore, this was associated with increased expression of innate immune receptors, including TLR and NOD (Deng et al., 2016). Quite an opposite pattern was observed for macrophages in our study, with *P. ciferrii* adhering more efficiently than *P. bovis*. This discrepancy may relate to the cell line-dependent susceptibility mentioned above (Deng et al., 2016; Shahid et al., 2017b), especially given that *P. bovis* is the major etiological agent of protothecal bovine mastitis (Arabe Filho et al., 2023).

The modest effect of MOI and time of incubation on the algal infection success aligns with previous findings. For instance, adhesion of *P. bovis* and *P. ciferrii* to bovine MECs increased steadily until 1.5 hours post-infection, at which point it reached a plateau (Deng et al., 2016). In a similar vein, no enhanced phagocytic uptake was observed at an extended time point upon exposure of *P. bovis* or *P. wickerhamii* to either murine or human macrophages (Haider et al., 2023). This illustrates a well-documented phenomenon of saturation of the binding capacity of host cells. In view of our results, the saturation of the surface receptors might have been reached at an MOI of 2 or lower and within two hours or less.

Although the ability of some *Prototheca* species to enter non-phagocytic cells, compels viewing this entry more as an invasion rather than mere internalization, adding a significant virulent attribute to the algae, this study does not provide mechanistic insights into this process. Still, certain clues are yielded by confocal and electron microscopy. Imaging studies revealed the intracellular localization of *Prototheca* in various developmental stages, including division. Captivatingly, despite the presence of the algae, the host’s intracellular architecture was not impaired. These observations are in line with previous findings, showing no ultrastructural alterations at early post-infection time points, with these changes, including cytoplasmic cavitation, mitochondrial swelling, pyknosis, and cytomembrane disruption, only becoming apparent in the later stages of infection (Shahid et al., 2017b; Shahid et al., 2017a). Importantly, the algae, while in the host cell, were observed outside the vacuoles, with no evidence of digestion, as guided by TEM analysis of infected macrophages. This suggests that *Prototheca* may escape from the phagosomes and persist in the host cytoplasm, as previously proposed for *Prototheca*, based on infection models involving phagocytic and non-phagocytic cells (Shahid et al., 2017b; Yang et al., 2020). A similar behavior is observed in intracellular opportunistic pathogens, such as *Cryptococcus neoformans* or *Candida albicans (*Casadevall, 2008; Flieger et al., 2018).

In conclusion, this study explores the early stages of *Prototheca* infection in a murine *in vitro* model. Three major findings emerge. First, host-pathogen interactions were strongly species-dependent, with pathogenic species (particularly *P. ciferrii*) showing higher adhesion and internalization than *P. stagnora*. Second, both adhesion and internalization were significantly more efficient in macrophages than in keratinocytes, and were only marginally affected by MOI and infection time. Third, microscopy-assisted observations indicated an extraphagosomal location of the algae, suggesting cytosolic escape as a strategy for their intracellular survival. Overall, this study advances understanding of the early events in the infectious life cycle of *Prototheca* algae and widens the path for future research on the pathobiology of these peculiar microorganisms.

## Data Availability

The original contributions presented in the study are included in the article/supplementary material. Further inquiries can be directed to the corresponding author.
